# Perioperative Outcomes and Trends in Transurethral Resection of Bladder Tumors with Photodynamic Diagnosis: Results from the GeRmAn Nationwide Inpatient Data Study

**DOI:** 10.3390/jcm13123531

**Published:** 2024-06-17

**Authors:** Nikolaos Pyrgidis, Marco Moschini, Lazaros Tzelves, Bhaskar K. Somani, Patrick Juliebø-Jones, Francesco Del Giudice, Laura S. Mertens, Renate Pichler, Yannic Volz, Benedikt Ebner, Lennert Eismann, Marie Semmler, Benjamin Pradere, Francesco Soria, Christian G. Stief, Gerald B. Schulz

**Affiliations:** 1 Department of Urology, University Hospital, Ludwig Maximilian University Munich, 81377 Munich, Germany; yannic.volz@med.uni-muenchen.de (Y.V.); lennert.eismann@med.uni-muenchen.de (L.E.); marie.semmler@med.uni-muenchen.de (M.S.);; 2 Department of Urology, Urological Research Institute, San Raffaele Scientific Institute, 20132 Milan, Italy; 3 2nd Department of Urology, Sismanoglio General Hospital of Athens, 15126 Athens, Greece; 4 Department of Urology, University of Hospital Southampton NHS Trust, Southampton SO16 6YD, UK; bhaskarsomani@yahoo.com; 5 Department of Urology, Haukeland University Hospital, 5009 Bergen, Norway; jonesurology@gmail.com; 6 Department of Urology, Sapienza Rome University, Policlinico Umberto I, 00161 Rome, Italy; francesco.delgiudice@uniroma1.it; 7 Department of Urology, Medical University Innsbruck, 6020 Innsbruck, Austria; ls.mertens@gmail.com (L.S.M.); renate.pichler@i-med.ac.at (R.P.); 8 Department of Urology, Comprehensive Cancer Center, Medical University of Vienna, 1090 Vienna, Austria; 9 Department of Surgical Sciences, University of Turin, Città della Salute e della Scienza, 10126 Turin, Italy

**Keywords:** transurethral resection of bladder tumors, bladder cancer, photodynamic diagnosis, perioperative outcomes, cohort study

## Abstract

**Background:** Photodynamic diagnosis (PDD) during transurethral resection of bladder tumor (TURBT) is guideline recommended, as it improves bladder cancer detection rates. However, the extent to which PDD is implemented in everyday clinical practice has not been thoroughly assessed. We aimed to evaluate the current trends and major perioperative outcomes of TURBT with PDD. **Methods:** The present study evaluated the GeRmAn Nationwide inpatient Data (GRAND) from 2010 (the year when PDD started to be coded separately in Germany) to 2021, which were made available from the Research Data Center of the German Bureau of Statistics. We undertook numerous patient-level and multivariable logistic regression analyses. **Results:** Overall, 972,208 TURBTs [228,207 (23%) with PDD and 744,001 (77%) with white light] were performed. Patients offered PDD during TURBT were younger (*p* < 0.001), presented fewer comorbidities (*p* < 0.001) and were discharged earlier from hospital (*p* < 0.001). PDD was associated with additional costs of about EUR 500 compared to white-light TURBT (*p* < 0.001). The yearly TURBT cases remained relatively stable from 2010 to 2021, whereas utilization of PDD underwent a 2-fold increase. After adjusting for major risk factors in the multivariate regression analysis, PDD was related to lower rates of transfusion (1.4% vs. 5.6%, OR: 0.29, 95% CI: 0.28 to 0.31, *p* < 0.001), intensive care unit admission (0.7% vs. 1.4%, OR: 0.56, 95% CI: 0.53 to 0.59, *p* < 0.001) and 30-day in-hospital mortality (0.1% vs. 0.7%, OR: 0.24, 95% CI: 0.22 to 0.27, *p* < 0.001) compared to white-light TURBT. On the contrary, PDD was related to clinically insignificant higher rates of bladder perforation (0.6% versus 0.5%, OR: 1.3, 95% CI: 1.2 to 1.4, *p* < 0.001), and reoperation (2.6% versus 2.3%, OR: 1.2, 95% CI: 1.1 to 1.2, *p* < 0.001). **Conclusions:** The utilization of PDD with TURBT is steadily increasing. Nevertheless, the road toward the establishment of PDD as the standard of care for TURBT is still long, despite of the advantages of PDD.

## 1. Introduction

Photodynamic diagnosis (PDD) during transurethral resection of bladder tumor (TURBT) is currently recommended for non-muscle-invasive bladder cancer [1,2]. PDD uses an intravesical photosensitizer, typically hexaminolevulinate, to cause tumors to fluoresce under blue light and guide TURBT [3]. Fluorescence-guided TURBT seems to be more sensitive than conventional white-light TURBT for the detection of non-muscle-invasive bladder cancer, mainly carcinoma in situ, offering better diagnostic accuracy and, in turn, reducing subsequent bladder cancer recurrences [4]. Failure to identify and remove the full extent of the tumor during TURBT may be the cause in up to 40% of all recurrences [5].

Even though PDD presents a higher sensitivity compared to white-light TURBT, it is also associated with lower specificity [6]. Hexaminolevulinate also accumulates in inflammatory tissues and causes non-cancerous areas of the urinary bladder to fluoresce. The latter may lead surgeons to resect these non-cancerous areas [7], and, in turn, increase postoperative complications such as the need for reoperation or perforation of the urinary bladder [8]. Available guidelines on non-muscle-invasive bladder cancer recommend the regular use of PDD at TURBT [9,10]. Nevertheless, real-world data assessing the extent to which PDD is implemented in everyday clinical practice are scarce [11]. Accordingly, high-volume studies assessing the baseline characteristics and perioperative complications of patients undergoing TURBT with PDD versus TURBT with white-light cystoscopy are lacking [12]. In this context, we aimed to assess the current trends and major perioperative outcomes of PDD during TURBT by performing the largest study in this domain.

## 2. Methods

### 2.1. GeRmAn Nationwide Inpatient Data (GRAND)

The GRAND study register is a nationwide German dataset that contains data on baseline characteristics, in-hospital procedures, and in-hospital outcomes from all patients who were admitted into a hospital in Germany between 2005 and 2021, except for those admitted for forensic, psychiatric, and military reasons. All German hospitals have to send to the corresponding Institute for the Hospital Remuneration System these in-hospital patient data to receive their remuneration. The data are then sent to the Research Data Center of the German Bureau of Statistics to be stored anonymized for research purposes. These data are coded according to the ICD-10-GM (International Statistical Classification of Diseases and Related Health Problems, 10th revision, German modification), as well as according to the OPS (German Procedure Classification).

For the purposes of the analysis for the present outcomes, access to the overall data of the GRAND study registry was gained through agreement with the Research Data Center of the German Bureau of Statistics (agreement: LMU-4710-2022). Following the existing legislation in Germany, approval by an institutional review board or written informed consent from the patient were not necessary. All analyses were undertaken, on our behalf, from the Federal Bureau of Statistics based on R codes (R statistical software, version 3.6.3) written by the present study group. Subsequently, the summary findings of these analyses were sent from the Research Data Center of the German Bureau of Statistics to our study group for additional evaluation (sources: Research Data Center of the German Bureau of Statistics, DRG Statistics 2005–2021, own calculations).

### 2.2. Selection Criteria and Outcomes

Given that PDD during TURBT has been coded in Germany since 2010, we included all hospitalized patients undergoing white-light (OPS code: 5-573.40) or PDD during TURBT (OPS code: 5-573.41) due to suspected bladder cancer (ICD code: C67 and D41.4). Patients undergoing TURBT for non-oncological reasons, as well as those undergoing surgery before 2010 were excluded from the present analysis. The primary outcomes of the present study were to evaluate the trends of PDD use in Germany. Secondary outcomes included the perioperative outcomes (intensive care unit admission, transfusion, 30-day mortality, bladder perforation and reoperation rates) of TURBT with PDD versus TURBT with white-light cystoscopy. Nevertheless, due to the inherent limitations of the GRAND, the oncological outcomes between TURBT with PDD and white-light cystoscopy could not be assessed.

### 2.3. Statistical Analysis

The available continuous variables were estimated as the median and interquartile range (IQR) and the available categorical variables as frequencies and proportions. The necessary comparisons between TURBT with PDD versus TURBT with white-light cystoscopy were undertaken with the Mann–Whitney and the chi-squared tests. We performed a multivariate logistic regression analysis to evaluate the perioperative outcomes (intensive care unit admission, transfusion, 30-day mortality, bladder perforation and reoperation rates) of TURBT with PDD versus TURBT with white-light cystoscopy. Based on clinical relevance, all models were adjusted for age, obesity, history of chronic kidney disease, hypertension, diabetes, benign prostatic hyperplasia, and active hematuria at the time of the operation. The odds ratio (OR) with its 95% confidence intervals (CI) were estimated for all logistic regression models and two-sided *p*-values < 0.05 were considered, for all analyses, to be statistically significant.

## 3. Results

### 3.1. Baseline Characteristics

A total of 972,208 TURBTs due to suspected bladder cancer were performed in Germany from 2010 to 2021. Patients undergoing TURBT had a median age of 74 (IQR: 65 to 80) years. Of these, 554,327 (57%) presented with hypertension, 123,396 (13%) with chronic kidney disease, 199,487 (21%) with diabetes and 131,570 (14%) with benign prostatic hyperplasia. The median hospital stay after TURBT was 4 (IQR: 3 to 6) days and the median hospital revenue for the operation was EUR 2393 (IQR: 2137 to 2846). Overall, 228,207 (23%) TURBTs were undertaken with PDD and 744,001 (77%) TURBTs with white-light cystoscopy. Patients offered PDD during TURBT in Germany were younger (*p* < 0.001), presented with fewer comorbidities (*p* < 0.001 for all baseline characteristics) and were discharged from the hospital earlier (*p* < 0.001). On the other hand, PDD was associated with additional costs of about EUR 500 compared to white-light cystoscopy (*p* < 0.001). All baseline characteristics of the selected patients are summarized in Table 1.

**Figure 1:**
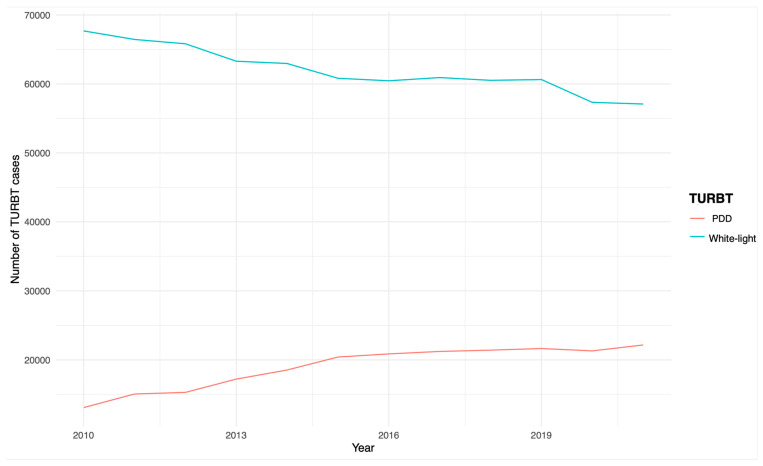
The annual trends of TURBT. PDD: photodynamic diagnosis; TURBT: transurethral resection of bladder tumor.

In Germany, the overall number of TURBTs due to suspected bladder cancer has remained relatively stable in recent years. In particular, 80,772 operations were performed in 2010 and 79,253 in 2021, with 2019 being the year with the most operations, with a total of 82,283. The use of PDD during TURBT has steadily increased during the last eleven years, with 13,085 cases performed in 2010 and 22,163 in 2021. On the contrary, TURBT with white light steadily decreased, with 67,687 cases performed in 2010 and 57,090 in 2021. The annual trends of TURBT are depicted in Figure 1.

### 3.2. Perioperative Outcomes after TURBT

In the multivariate analysis, after adjusting for age, obesity, history of chronic kidney disease, hypertension, diabetes, benign prostatic hyperplasia, and active hematuria at the time of the operation, PDD during TURBT was related with significantly lower rates for transfusion (1.4% versus 5.6%, OR: 0.29, 95% CI: 0.28 to 0.31, *p* < 0.001), intensive care unit admission (0.7% versus 1.4%, OR: 0.56, 95% CI: 0.53 to 0.59, *p* < 0.001) and 30-day in-hospital mortality (0.1% versus 0.7%, OR: 0.24, 95% CI: 0.22 to 0.27, *p* < 0.001) compared to TURBT with white-light cystoscopy. On the contrary, PDD during TURBT was related with clinically insignificant higher rates for urinary bladder perforation (0.6% versus 0.5%, OR: 1.3, 95% CI: 1.2 to 1.4, *p* < 0.001), and reoperation during hospital stay (2.6% versus 2.3%, OR: 1.2, 95% CI: 1.1 to 1.2, *p* < 0.001). The multivariate logistic regression is presented in Table 2.

## 4. Discussion

The results of the available GRAND analysis demonstrate that less than one-fourth of all TURBTs in Germany between 2010 and 2021 were performed with PDD. Although this proportion has steadily increased in the past few years, it remains low. Importantly, the overall number of TURBT cases performed in Germany remained relatively stable from 2010 to 2021. It should be stressed that these findings demonstrate that patients receiving PDD during TURBT present better baseline characteristics. Based on the previous notion, patients undergoing PDD during TURBT presented lower odds of transfusion, admission to the intensive care unit and 30-day in-hospital mortality. On the contrary, PDD during TURBT was associated with higher odds of urinary bladder perforation and reoperation compared to white-light TURBT.

Our findings indicate that TURBT with PDD is performed in healthier patients and, thus, leads to lower odds of transfusion, admission to the intensive care unit and 30-day in-hospital mortality. Given that randomized controlled studies and relevant meta-analyses demonstrate that PDD during TURBT displays similar rates of serious surgical complications and perioperative mortality [6,13,14] compared to white-light TURBT, it seems that the findings of our analysis may simply be the result of residual confounding with major patient comorbidities. Based on the previous notion, a combined analysis of the randomized controlled trials used for the approval and long-term postmarketing surveillance of PDD suggests that PDD is safe and is not associated with further complications compared to white-light TURBT [12]. Nevertheless, our findings indicate that, after adjusting for major risk factors, PDD during TURBT may increase the odds of urinary bladder perforation and reoperation by 30% and 20%, accordingly. The latter may be explained by the additional resections or biopsies from PDD-positive areas of the urinary bladder [15]. Still, it should be noted that the overall rates of complications were lower than other relevant studies [16,17,18,19]. Considering that the incidence of major complications in the present study were relatively low, the statistically significant differences between both groups, may not be of clinical importance.

It should be stressed that the proportion of annual TURBT cases that were performed with PDD is relatively low in Germany, where PDD was developed and established [20,21]. Nonetheless, this proportion is significantly higher compared to that in the USA. In particular, from 2016 to 2020, 1.2% of all index TURBTs were performed with PDD, although PDD during TURBT is also recommended from the AUA guidelines [10]. Notably, in centers with PDD equipment, only about 10% of all index TURBTs are performed with PDD. To complicate things further, the use of PDD seems to have plateaued in recent years [11]. Moreover, hospital-level availability of PDD technology seems to be less than 5%, even though the upgrading from pre-existing TURBT equipment to PDD compatible equipment costs about EUR 8000 [22]. Accordingly, the cost of hexaminolevulinate is approximately EUR 300–500 per patient [23]. Nevertheless, analyses on the cost-effectiveness of the procedure suggest that PDD is clearly associated with significant long-term cost savings [24]. More specifically, PDD may result in the removal of all tumors during TURBT, as well as in earlier diagnosis of high-risk tumors, preventing bladder cancer recurrence and progression in some patients and leading, in turn, to fewer and less serious procedures in the long term [25]. Therefore, the additional costs of TURBT with PDD are completely covered by healthcare insurance in Germany [22,23].

Although we present, to the best of our knowledge, the largest study on trends and perioperative outcomes after TURBT, these findings were tempered by some important limitations that need to be considered. The GRAND study register is based on retrospective, administrative data that are prone to misclassifications and coding errors, despite the fact that these data present a high degree of accuracy and are regularly controlled by independent physician task forces from healthcare insurances. It should be stressed that major perioperative information such as patients’ laboratory findings, operative time, oncological status (histology findings, TNM classification, and surgical margins) as well as long-term complications, or readmission rates after hospital discharge are not available in the GRAND study. Accordingly, no information on the type of surgeon (in training or senior), type of hospital (academic or non-academic) and on the proportion of hospitals performing resections with PPD could be provided. Moreover, a long-term and in-depth cost analysis could not be performed. Importantly, recurrence and progression rates after TURBT with PDD versus white-light cystoscopy, re-TURBT rates, as well as history of previous or need for subsequent intravesical instillation therapy, are not available in the GRAND study. It was also beyond the present analysis to compare PDD with other new methods of tumor visualization. It should be also noted that our results were made available from nationwide German data and, therefore, they cannot be easily applied to other medical healthcare systems around the world. Nevertheless, aiming to overcome these important limitations, we undertook multiple analyses that may be valuable for proper preoperative patient counseling.

## 5. Conclusions

The present high-volume, real-world data from Germany demonstrate a steadily increasing utilization of PDD during TURBT for suspected bladder cancer. Nevertheless, it should be highlighted that the road to establishing PDD as the standard of care for TURBT is still long, given that, to date, the majority of all TURBTs are performed without PDD. Considering that PDD increases the diagnostic accuracy of TURBT and that it presents specific significant advantages, the additional EUR 500 per patient that are needed for its everyday use (combined with the EUR 8000 cost of its installation) do not document its low implementation in Germany and in other high-income countries. Based on our analyses, patients offered TURBT present favorable baseline characteristics and, in turn, the operation with PDD is associated with better perioperative outcomes compared to white-light TURBT. Moreover, PDD was associated, as expected, with higher, but clinically insignificant, bladder perforation and reoperation rates. Overall, our findings demonstrate a concerning underutilization in the adoption of an evidence-based and guideline-supported diagnostic modality, which may further improve bladder cancer outcomes if it starts being widely implemented.

## Figures and Tables

**Table 1:** The baseline characteristics of patients undergoing transurethral resection of bladder tumor. Variables are available as median and interquartile range or frequencies and proportions. The Mann–Whitney test was undertaken for all comparisons between continuous variables and the chi-squared test was undertaken for all comparisons between categorical variables. The bold *p*-values indicate statistically significant differences between the two groups. PDD: photodynamic diagnosis.

Characteristic	Overall, *n* = 972,208	PDD, *n* = 228,207	No PDD, *n* = 744,001	*p*-Value
**Age (years)**	74 (65–80)	72 (64–79)	74 (66–81)	**<0.001**
**Hospital stay (days)**	4 (3–6)	3 (2–5)	4 (3–6)	**<0.001**
**Hospital revenues (Euros)**	2393 (2137–2846)	2791 (2663–2962)	2230 (2091–2573)	**<0.001**
**Diabetes**	199,487 (21%)	43,136 (19%)	156,351 (21%)	**<0.001**
**Chronic heart failure**	57,553 (5.9%)	9176 (4.0%)	48,377 (6.5%)	**<0.001**
**Chronic obstructive pulmonary disease**	83,410 (8.6%)	17,460 (7.7%)	65,950 (8.9%)	**<0.001**
**Chronic kidney disease**	123,396 (13%)	22,046 (9.7%)	101,350 (14%)	**<0.001**
**Cerebrovascular disease**	23,915 (2.5%)	3993 (1.7%)	19,922 (2.7%)	**<0.001**
**Dementia**	24,040 (2.5%)	2777 (1.2%)	21,263 (2.9%)	**<0.001**
**Hypertension**	554,327 (57%)	126,500 (55%)	427,827 (58%)	**<0.001**
**Benign prostatic hyperplasia**	131,570 (14%)	28,675 (13%)	102,895 (14%)	**<0.001**

**Table 2:** The multivariate logistic regression analysis in terms of transfusion, ICU admission, 30-day mortality, bladder perforation and reoperation rates after white-light and PDD TURBT. All models are adjusted for age, obesity, history of chronic kidney disease, hypertension, diabetes, benign prostatic hyperplasia, and hematuria at the time of the operation. The bold cells indicate statistically significant *p*-values. ICU: intensive care unit; OR: odds ratio; PDD: photodynamic diagnosis; TURBT: transurethral resection of bladder tumor.

	Transfusion			ICU Admission			30-Day Mortality			Bladder Perforation			Reoperation		
TURBT	Events	OR	*p*-Value	Events	OR	*p*-Value	Events	95% CI	*p*-Value	Events	OR	*p*-Value	Events	OR	*p*-Value
White-light	41,912 (5.6%)	—	—	10,624 (1.4%)	—	—	5409 (0.7%)	—	—	3637 (0.5%)	—	—	16,861 (2.3%)	—	—
PDD	3122 (1.4%)	0.29 (0.28, 0.31)	**<0.001**	1582 (0.7%)	0.56 (0.53, 0.59)	**<0.001**	310 (0.1%)	0.24 (0.22, 0.27)	**<0.001**	1338 (0.6%)	1.3 (1.2, 1.4)	**<0.001**	6045 (2.6%)	1.2 (1.1, 1.2)	**<0.001**

## Data Availability

All data used in this work are stored in anonymized form at the German Federal Statistical Office.
